# The Importance of the Microbiome in Critically Ill Patients: Role of Nutrition

**DOI:** 10.3390/nu11123002

**Published:** 2019-12-07

**Authors:** Rocio Moron, Julio Galvez, Manuel Colmenero, Per Anderson, José Cabeza, Maria Elena Rodriguez-Cabezas

**Affiliations:** 1 Servicio Farmacia Hospitalaria, Hospital Universitario Clínico San Cecilio, 18016-Granada, Spain; rmoronr@gmail.com (R.M.); jose.cabeza.sspa@juntadeandalucia.es (J.C.); 2 Instituto de Investigación Biosanitaria (ibs.GRANADA), 18012 Granada, Spain; manuel.colmenero.sspa@juntadeandalucia.es (M.C.); per.anderson@ibsgranada.es (P.A.); merodri@ugr.es (M.E.R.-C.); 3 Department of Pharmacology, CIBER-ehd, Center of Biomedical Research (CIBM), University of Granada, 18071 Granada, Spain; 4 Servicio de Medicina Intensiva, Hospital Universitaro Clinico San Cecilio, 18016 Granada, Spain; 5 Servicio de Análisis Clínicos e Inmunologia, UGC Laboratorio Clínico, Hospital Universitario Virgen de las Nieves, 18014 Granada, Spain

**Keywords:** critically ill patient, microbiome, nutrition, probiotics, prebiotics, synbiotics, fecal microbiota transplantation

## Abstract

Critically ill patients have an alteration in the microbiome in which it becomes a disease-promoting pathobiome. It is characterized by lower bacterial diversity, loss of commensal phyla, like Firmicutes and Bacteroidetes, and a domination of pathogens belonging to the Proteobacteria phylum. Although these alterations are multicausal, many of the treatments administered to these patients, like antibiotics, play a significant role. Critically ill patients also have a hyperpermeable gut barrier and dysregulation of the inflammatory response that favor the development of the pathobiome, translocation of pathogens, and facilitate the emergence of sepsis. In order to restore the homeostasis of the microbiome, several nutritional strategies have been evaluated with the aim to improve the management of critically ill patients. Importantly, enteral nutrition has proven to be more efficient in promoting the homeostasis of the gut microbiome compared to parenteral nutrition. Several nutritional therapies, including prebiotics, probiotics, synbiotics, and fecal microbiota transplantation, are currently being used, showing variable results, possibly due to the unevenness of clinical trial conditions and the fact that the beneficial effects of probiotics are specific to particular species or even strains. Thus, it is of great importance to better understand the mechanisms by which nutrition and supplement therapies can heal the microbiome in critically ill patients in order to finally implement them in clinical practice with optimal safety and efficacy.

## 1. Introduction

The microbiome has been intensely studied and the understanding of its metabolic and immunological functions has had remarkable advances. The disruption of the microbiome homeostasis, known as “dysbiosis” or “pathobiome”, can be as important as the host genetics for the development of various conditions, such as inflammatory bowel disease, obesity, diabetes, or cardiovascular disease. In critically ill patients, who are affected by a life-threatening multisystem process that can result in significant morbidity or mortality [1], many factors can contribute to the development of a pathobiome, including intrinsic factors, like genetics or age, and those that can be manipulated by either the human host or medical interventions, such as diet, geographic location, or drug therapy [2,3,4]. Lately, special attention has been paid to the relationship between nutrition and the microbiome, but more data is needed to understand which nutrients participate in the maintenance of the microbiome homeostasis in health and disease, and which interventions could help to recover this homeostasis during and after critical illness, like nutritional supports or the use of probiotics, prebiotics, and fecal transplantation. The aim of this review is to present the current knowledge about the role of the microbiome in critically ill patients and the modulatory role of nutrition, which can determine their evolution and the efficacy of the current therapeutic strategies.

## 2. The Gut Microbiome

The human gut contains more than 1000 different microbial species that collectively encode over 100 times more genes than the human genome [5]. In healthy humans, the intestinal microbiome is composed of members of the three domains of life—bacteria, archaea, and eukaryotes, although the bacterial community is the most abundant and heterogeneous. Nine different bacterial phyla have been reported, with Bacteroidetes and Firmicutes being the most dominant members [6,7,8].

It is difficult to characterize all the populations since many of them cannot be grown in vitro. However, recent advances in the culture methods for "non-cultivable" human microbes have revealed a whole spectrum of new species and bacterial taxa [9]. Moreover, techniques such as 16S rRNA and shotgun metagenomic sequencing have opened a new area of research, allowing for the identification of complex populations of bacteria, and their effects on health and disease [10,11].

Currently, the microbiome is recognized as a separate organ, considering its diverse roles in metabolism, immune system development and host defence against pathogens, and intestinal maturation and functions, such as nutrient uptake and metabolism, mucosal barrier, enteric nervous system, and motility [12,13,14,15]. Moreover, numerous host genes appear to be specifically altered in response to certain members of the microbiome, showing the importance of the microbial composition for the body’s responses [16,17]. This interaction between the immune system and the microbiome has been revealed using germ-free mice, which lack all commensal bacteria. They fail to develop a mature immune system and are more susceptible to viruses, bacteria, and pathogenic fungi [18,19], highlighting the importance of the microbiome for the development and function of the immune system.

## 3. The Gut Microbiome in Critically Ill Patients

### 3.1. Changes in the Gut Microbiota in Critically Ill Patients

The intestine has long been hypothesized as "the engine" of critical illness, but its clinical importance needs to be better defined. The gut microbiome is severely altered in multiple disease states, including critical illness, where the health-inducing microbiome becomes a disease-promoting pathobiome that makes the patient more vulnerable to nosocomial infections, sepsis, and multiple organ failure [20]. So far, only a few studies have analyzed the gut microbiome in critically ill patients, and they have confirmed a state of dysbiosis [21,22,23]. Moreover, recent studies in intensive care unit (ICU) patients observed a gradual worsening of the dysbiosis during their stay in the ICU [22,24,25,26]. The most relevant changes in the microbiome can be seen in the largest study to date that examined the sequencing of the 16S rRNA gene from multiple body sites (skin, oral, and feces) from 115 ICU patients and compared it with 1242 healthy volunteers [22]. At the intestinal level there was a low prevalence of the Firmicutes and Bacteroidetes phyla, and a greater richness of Proteobacteria in comparison to healthy individuals. At the genus level, there was a lower prevalence of key commensal genera (such as *Faecalibacterium*—an anti-inflammatory organism, *Blautia,* and *Ruminococcus*), and in some cases, an overgrowth (over 50% relative abundance) of genera with pathogenic properties, such as *Escherichia*/*Shigella*, *Salmonella*, *Enterococcus*, *Clostridium difficile,* or *Staphylococcus* [21,22,23,24,27]. It has been proposed that changes in the *Firmicutes/Bacteroidetes* ratio can predict patient outcome [24], although further work is required to validate these findings.

Overall, critically ill patients admitted to the ICU present a gut microbiome characterized by lower bacterial diversity and large inter-individual variation. A study of 14 ICU patients also reported the emergence of ultra-low-diversity communities in 35% of patients who only presented one to four bacterial taxa [27]. In previous studies, low microbial diversity has been associated with an increased risk of mortality [28,29], and the domination of certain pathogens have been identified as an independent risk factor for adverse outcomes [30,31,32].

**Figure 1:**
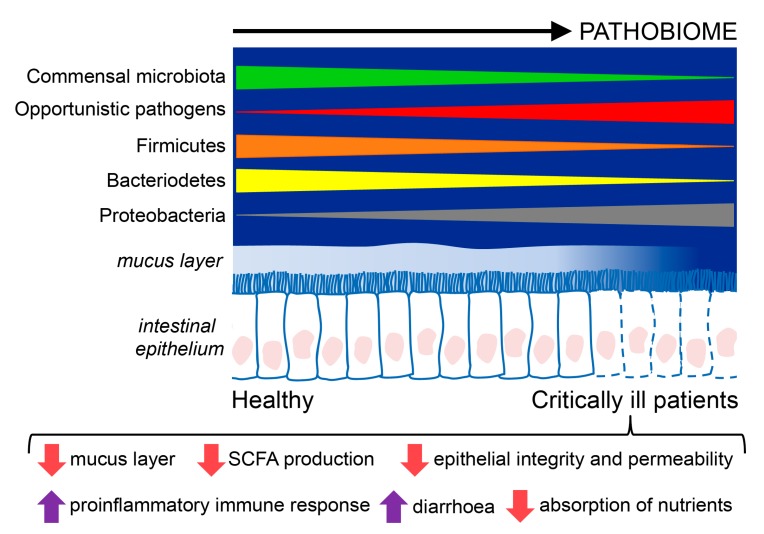
Composition and functions of the intestinal microbiome in critically ill patients compared to healthy individuals. Critically ill patients exhibit an intestinal disease-promoting microbiome or pathobiome. This pathobiome is characterized by a lower prevalence of the Firmicutes and Bacterioidetes phyla, and a higher prevalence of the Proteobacteria phyla, in contrast to healthy individuals. Furthermore, the intestinal epithelium is altered in critically ill patients, showing reduced reperfusion, that could lessen the hydrophobicity of the mucus layer and favor the translocation of pathogens through gaps between the epithelial cells, and epithelial apoptosis, resulting in poor absorption of nutrients, diarrhea, loss of fecal energy, and lower production of short chain fatty acids (SCFA).

Considering all the studies on critically ill patients, Proteobacteria is the dominant phyla, and Firmicutes is reduced, whereas Enterococcus, Staphylococcus, and Enterobacter are increased in septic patients. In these patients with sepsis, the focus often lies on the identification of a single pathogen as the causative agent. However, there is an increasing belief that most infections have “polymicrobial” phenotypes that depend on the microbiome status of the patient. Thus, the initial state of the microbiome can determine both the susceptibility to infection [33] and its severity [34]. The composition and functions of the intestinal microbiome of critically ill patients and healthy humans are summarized in Figure 1.

Furthermore, migration of microorganisms between the intestinal and pulmonary microbiome has been reported in critically ill patients. A recently published study highlights the impact of the gut microbiome on the pulmonary microbiome. It was observed that the pulmonary microbiome in both murine sepsis and human acute respiratory distress syndrome (ARDS) was enriched with bacteria associated with the intestine. An operative taxonomic unit of Bacteroides was detected in the bronchoalveolar fluid (BAL) samples from 41% of patients with ARDS compared to 3% in healthy patients. Moreover, the systemic and alveolar levels of tumor necrosis factor (TNF)-α in patients with ARDS were markedly increased by the presence of organisms derived from the intestine in the BAL. However, the precise route by which the intestinal microorganisms reached the lungs of the mice with sepsis has not been identified [35].

### 3.2. Modulators of the Microbiome in Critical Illness

The alteration of the microbiome in critically ill patients is multicausal. Critical disease leads to profound modifications in the gut microbiome, caused by general alterations in the host environment [36,37] including enhanced virulence of the bacteria due to the expression of ancestral or newly acquired genes [38].

In addition, many treatments administered to patients in the ICU, like antibiotics, proton pump inhibitors, vasopressors, and opioids, produce harmful effects outside their target organ, which directly affect the microbiome. The most significant alterations are probably related to antibiotic treatments since they indiscriminately ablate the commensal microbiome, favoring the intrusion of secondary pathogenic microorganisms and the enrichment of antibiotic resistance genes [39]. Thus, antibiotic therapies may aggravate the alteration of the microbiome caused by the different pathologies. In fact, the use of antibiotics in ICUs is very frequent, with 71% of the patients receiving antibiotic treatment, according to data from the Centers for Disease Control and Prevention in the United Sates [40]; although it is estimated that 35% of the antibiotic regimens are unnecessary, according to the latest recommendations. As a result, the increase in mortality and morbidity associated with these alterations leads to an additional increase in the cost and care related to the ICU.

Besides, the inappropriate use of antibiotics is considered to be responsible for the increasing emergence of multidrug-resistant bacteria (MDR), and it is important to consider that nosocomial infections represent an additional complication in critically ill patients. The incidence of MDR infections is escalating rapidly all over the world [41]. In fact, a recent publication calculated that infections with antibiotic-resistant *Clostridium difficile* occur in more than 450,000 patients per year, in the United States [41]. In addition, MDR infections are increasingly lethal for hospitalized patients. It is calculated that *C. difficile* contributes to more than 30,000 deaths per year in the United States [41,42]. Consequently, the implementation of new antibiotic drugs has not significantly improved the survival to infectious diseases in developed countries, but has instead contributed to the emergence of increasingly aggressive MDR organisms.

**Figure 2:**
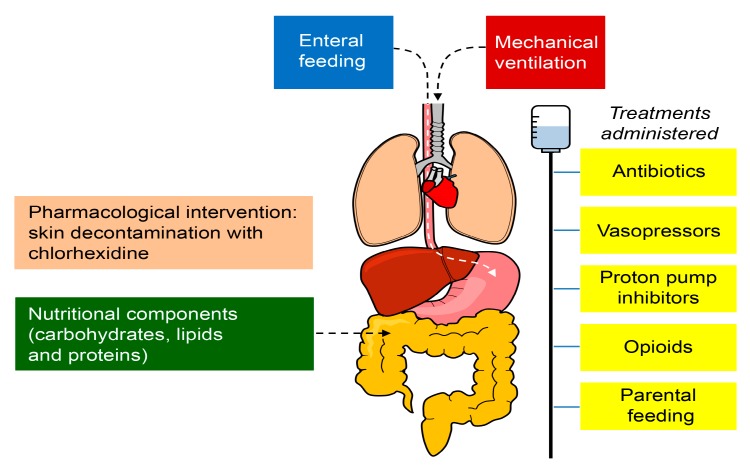
Factors that may alter the microbiome in critically ill patients in the ICU. The treatments administered to patients in the ICU, including antibiotics, proton pump inhibitors, vasopressors, and opioids, produce harmful effects outside their target organ, which directly affect the microbiome. The nutritional components (carbohydrates, lipids, and proteins) and the route of administration (enteral/parenteral) might also alter the health of the microbiome. The pharmacological interventions can modify the specific conditions of the body site (for example, skin decontamination with chlorhexidine) and invasive procedures may impair the natural barrier mechanisms (e.g., endotracheal intubation and intravascular catheters), facilitating the access and proliferation of microbes.

Moreover, nutrition is another key factor for the gut microbiome homeostasis since it primarily depends on the availability of enteral nutrients for survival. Thus, the nutritional components (carbohydrates, lipids, and proteins) and the route of administration (enteral/parenteral) might also alter the health of the microbiome [43,44,45]. In addition, pharmacological interventions can modify the specific conditions of the body site (for example, skin decontamination with chlorhexidine) and invasive procedures may alter the natural barrier mechanisms (e.g., endotracheal intubation, intravascular catheters) that could facilitate the access and proliferation of microbes [46]. The factors that may alter the microbiome are shown in Figure 2. Therefore, the impact of ICU care on the microbiome should be further explored.

### 3.3. Epithelial Alterations and Intestinal Hyperpermeability in Critically Ill Patients

Critical illness induces hyper-permeability of the gut barrier that begins as early as one hour after the onset of sepsis or trauma and lasts for at least 48 h [47,48]. Mucus also plays a crucial role in the defence of the host by preventing bacteria and digestive enzymes from contacting the intestinal epithelium as a result of mucus hydrophobia that reduces the absorption of toxic molecules. In critical disease, the mucus layer is affected, which induces the dysfunction of epithelial cells. Actually, in these patients, it is very common to find a reduced intestinal reperfusion that can lessen the hydrophobicity of the mucus layer and alter the intestinal permeability [49].

One of the mechanisms responsible for the epithelial defects in critically ill patients could be the impairment of short chain fatty acids (SCFA) production. One important metabolic function of the gut microbiome is the fermentation of dietary fiber and production of SCFA, including butyrate, which serves as the primary energy source for the colonic epithelium and preserves its integrity [50]. During sepsis, a rapid and persistent fall in the concentration of SCFA takes place [51] and as a consequence, the mucosal epithelial barrier is impaired due to epithelial apoptosis resulting in poor absorption of nutrients, diarrhea, loss of fecal energy, and pathogen translocation [22,23]. However, it has been described in a graft versus host disease mouse model that when bacterial strains, which are capable of producing large amounts of SCFA, are ingested, the severity of the disease decreases. This is explained by the high intestinal concentrations of butyrate that improve the epithelial barrier by enhancing intercellular junctions and decreasing cell apoptosis [52].

### 3.4. Relevance of the Gut Microbiome in Critical Illness

Considering the microbiome as an internalized organ with important physiological functions, it is evident that its alteration might be as harmful as other “organ failures” in ICU patients. The possible damage could be caused by both the loss of “organ” function and also the aberrant physiology replacing its function. In this context, the “lost organ” is the commensal microbial community that helps to metabolize drugs, nutrients, and hormones, modulate immune responses, and maintain the mucosal barrier homeostasis. By losing commensal microbes, the host also loses protection against invading pathogens by different mechanisms. The gut microbiome is the main activator of the host immunity against infections, which involves innate (stimulation of granulopoiesis, production of antimicrobial peptides (bacteriocins), and nutrient source competition) [39,50] and adaptive (regulation and differentiation of Th17 cells) mechanisms [53]. The “aberrant physiology” is represented by emerging pathogens that dominate microbial communities and cause dysregulated inflammatory responses, excessive inflammation leading to multiple organ dysfunction, and eventually, immune depletion due to the loss of specific microbial signals necessary for the maintenance of normal T cell function in the gut, that could facilitate the emergence of super-infections [54] and ultimately sepsis [55].

Besides, in healthy individuals, bacteria rarely express virulence genes, while in situations of stress when resources are limited, like in the case of critical illness, bacteria can express ancestral and newly acquired resistance genes that could lead to bacterial invasion, and in turn, generate a maladaptive host response [20]. Thus, nowadays, the concept of "good" and "bad" bacteria is considered too simplistic since bacteria can alter their own virulence depending on the host factors, i.e., identical bacterial species can be adaptive or maladaptive depending on the clinical situation [37]. Additionally, pathogens have a greater power to compete with the commensal bacteria, which may be favored by the decrease in transit time, lack of nutrition, and use of antibiotics [5].

Moreover, it is known that commensal bacteria are involved in the regulation of the immunological properties of CD4+ T cells, possibly through SCFA production [51], although the exact mechanisms remain undiscovered [56]. Hence, a significant loss of protective anaerobes in fecal samples has been observed in patients with severe sepsis [25,29], which could indicate that the pathobiome could be capable of manipulating and deregulating the immune system in critically septic and diseased patients [29]. In this regard, it has been reported in a model of murine polymicrobial sepsis that opioid treatment leads to the selective translocation into the circulation and systemic spread of Gram-positive intestinal microorganisms which induce pro-inflammatory effects mediated by the production of interleukin (IL)-6 and IL-17A [36]. Likewise, it has also been described that the function and aging of neutrophils, which act as the first line of cellular defence, is also regulated by the microbiome during sepsis [57,58]. Considering the above, it could be plausible that particular therapeutic interventions in the altered microbiome could improve the barrier, immune, and organ functions, as well as the prognosis of sepsis.

Hence, recent preclinical data derived from animal models suggest that the intestinal microbiome plays a protective role in the host defence against sepsis [34,58,59]. In murine models of Gram-positive and Gram-negative pneumosepsis, it has been shown that antibiotics can induce the disruption of the gut microbiome, which increases inflammation and bacterial spread [34,59]. Similarly, data from ICU patients indicate that the loss of microbiome diversity implies an increased length of stay in the ICU, which further highlights the potential clinical relevance of the intestinal microbiome for critically ill patients [22].

## 4. Nutrition of the Critically Ill Patient 

Medical nutritional therapy in critically ill patients is a challenge due to the great heterogeneity among patients and the variable duration of the acute phase of disease, firstly characterized by hemodynamic instability and a severe increase in catabolism that later progresses to a period of muscle wasting and stabilization of the metabolic alterations [60]. The management of critically ill patients and the outcome of disease could be notably improved by monitoring their metabolic profile (protein-energy malnutrition, lipidome, and the levels of glucose, insulin, vitamin D_3_, and other micronutrients) [61,62] and implementing an individualized nutritional treatment. There are many guidelines for the care of critically ill patients, but the supporting studies lack external validity due to the heterogeneity of patients, resulting in no general agreement in the nutritional recommendations. Thus, further investigation is needed to achieve a consensus to guide clinicians [63,64].

Many studies have shown that the lack of enteral nutrition, which is very frequent in the ICU, may alter the intestinal microbiome composition and weaken the epithelial barrier function, predisposing it to bacterial translocation, which is also associated with septic complications [65,66].

For instance, the production of butyrate, the main energy source for intestinal epithelial cells, would be compromised since it is produced by the microbiome fermentation of dietary fibers in the large intestine. Recent experimental studies have demonstrated how this vicious cycle begins and that the gastrointestinal tract responds with an elaborate system of regulatory mechanisms that can be altered when enteral nutrients are absent [67].

“The ESPEN guidelines” have been published in order to offer the best medical nutritional therapy to ICU patients [60]. They recommend medical nutritional therapy for all patients admitted to the ICU, and particularly to those staying for more than 48 h, including administration of oral nutritional supplements, enteral nutrition, and parenteral nutrition. Moreover, they encourage the use oral nutrition, but when it is not possible, enteral nutrition should be initiated within 48 h.

Enteral nutrition has greater benefits in the critically ill patient, making it the preferred modality in patients with a functioning gastrointestinal tract. The main benefit lies in the activation of luminal detection mechanisms, which stimulate and modulate the cellular activities of the mucosa. However, many critically ill patients cannot receive enteral nutrition due to intolerance problems or clinical conditions where enteral nutrition is contraindicated according to the European Society of Intensive Medicine (ESCIM) [68] (Table 1).

In these patients, parenteral nutrition has been a life-saving supportive treatment. Although early studies associated parenteral nutrition with high rates of complications, mainly of infectious nature, current guidelines recommend the implementation of parenteral nutrition, in the case of oral and enteral nutrition contraindications, within three to seven days [60].

### 4.1. Diet Composition: Effect on Gut Microbiome

Numerous studies have shown a great impact of diet on the composition of the intestinal microbiome. Even short-term exposure to diets that are very rich in a specific type of macronutrient promotes the selection of bacteria with the genetic capacity to metabolize the components of the diet and survive within that particular environment. For example, an animal-based diet is associated with the proliferation of bile-tolerant microorganisms and the reduction of bacteria that metabolize plant polysaccharides [45]. The analysis of gut microbial communities has demonstrated the existence of two well-defined enterotypes, dominated by *Bacteroides* and *Prevotella* [69], which are associated with Western diets based on protein and animal fat consumption, and carbohydrate-based diets, respectively [43]. In addition, animal-based diets induce the proliferation of the Bacteroidetes and Actinobacteria phyla, while reducing the abundance of Firmicutes and Proteobacteria. However, Firmicutes and Proteobacteria are positively associated with high-fiber intake, and Bacteroidetes and Actinobacteria show the opposite correlation. Besides, many dietary ingredients, different from macronutrients, can affect the gut microbiome. Enteral diets may contain diverse synthetic dietary emulsifiers and preservatives, including carboxymethyl cellulose, soy lecithin, gum arabic, soy polysaccharide, and various glycerol derivatives that have been linked to intestinal dysbiosis [70]. In this regard, Chassaing et al [71], found that two commonly used dietary emulsifiers, carboxymethyl cellulose and polysorbate-80, caused microbial instability of the gut microbiome in mice, characterized by an increase in the Verrucomicrobia and Proteobacteria phyla. Nevertheless, more studies are needed to properly define the effects of different types of enteral nutrition on the intestinal microbiome.

### 4.2. Enteral versus Parenteral Nutrition: Effect on Gut Microbiome

The latest evidence suggests that the way of providing nutritional therapy (i.e., enteral versus parenteral) is essential as it affects the microbiome differently [67]. It has been reported that parenteral nutrition in mice alters the composition of the intestinal microbiome, inducing an increase in Proteobacteria [34].

Therefore, starvation and parenteral nutrition are associated with a loss of bacterial diversity that may alter the microbiome interaction with the host immune system, and the capacity to control the growth of more potential pathogenic bacteria like *E. coli*, *Salmonella*, *Yersinia*, and *Helicobacter* o *Vibrio*, which might favor the appearance of infections as well as the increased expression of pro-inflammatory cytokines in the gut mucosa and loss of barrier function [72].

These alterations of the intestinal mucosa may be due to different mechanisms—(i) a decrease in the expression of alkaline phosphatase, a brush-border enzyme in enterocytes [73,74,75]; and (ii) activation of toll-like receptors by Gram-negative bacteria lipopolysaccharides that leads to a dysfunction of the intestinal barrier, up-regulation of TNF-α receptors (TNFR) in epithelial cells that decreases the expression of cytoskeletal proteins (claudins and occludins), and an alteration of epithelial occluding junctions, which allows bacterial translocation [76].

Moreover, using a human endotoxemia model, Lubbers et al. [77] showed that enteral nutrition exerted an anti-inflammatory effect, reflected by decreased serum levels of the pro-inflammatory cytokines, TNF-α and IL-6, and an elevated concentration of the anti-inflammatory cytokine, IL-10. Accordingly, mice receiving total parenteral nutrition exhibited an increased expression of interferon-γ in the intestinal epithelium, which was associated with the loss of intestinal barrier function, which could be partially mitigated by the administration of IL-10 [78]. However, in critically ill patients, it is difficult to find an association between the nutrition route and blood levels of pro- and anti-inflammatory cytokines. Notwithstanding, a relevant study in pediatric patients with critical illness comparing enteral and parenteral nutrition reported that high levels of IL-10 were independently associated with mortality in those patients receiving parenteral nutrition [79].

In the case of enteral nutrition, studies in mice have shown that the gut microbiome is dominated by Firmicutes, while in its absence, the main phyla are Proteobacteria, Bacteroidetes, and Verrucomicrobia. The mechanisms involved in these changes are not well known but the selection of potentially pathogenic bacteria more resistant to the lack of luminal nutrients together with factors within the host could participate [80]. Indeed, Firmicutes may be more dependent on carbohydrates and, thus, less competitive than other phyla in starvation states.

Interestingly, it has been reported in animal models that these alterations in the gut microbiome and the intestinal function can be reverted when enteral nutritional supplementation (at least 20%) is introduced [75].

Data regarding the changes in the microbiome of humans receiving parenteral nutrition are limited. However, studies performed in newborns receiving parenteral nutrition have reported progressive changes in their microbiome with a significantly lower abundance of Bacteroidetes and a greater abundance of Verrucomicrobia in comparison with the controls [81]. In addition, the enrichment of *Clostridium* species was observed [82,83].

### 4.3. Strategies to Modulate the Gut Microbiome in Critically Ill Patients

In summary, the presented data clearly suggest that the modulation of the microbiome in ICU patients could improve their prognosis. Importantly, restoration of the antibiotic-induced dysbiosis would also have the potential to reduce the risk of infections [84]. In this regard, it has been reported that therapies based on the use of prebiotics, probiotics, or synbiotics (combinations of probiotics and prebiotics) can reduce the ubiquitous disruption of the healthy microbiome that occurs in critical illness, improving the restoration of the microbiome, thus limiting the appearance of infections and ultimately accelerate recovery [40].

#### 4.3.1. Use of Prebiotics in Critically Ill Patients

Prebiotics are key supplements, which promote the maintenance of gut microbiome homeostasis. Fiber-rich diets show beneficial effects on the intestinal barrier integrity, preventing pathogen translocation in models of sepsis [85,86].

Although some prebiotics selectively promote the growth of fecal *Bifidobacterium* in healthy individuals, this has not been observed in critically ill patients [87]. A recent prospective cohort study on enterally=fed ICU patients who were administered with high soluble fiber reported a relative abundance of SCFA-producing bacteria, including *Faecalibacterium prausnitzii, Eubacterium rectale, Ruminococcus, Blautia, Coprococcus,* and *Roseburia*, improved abdominal distension, and showed no increase in diarrhea [88].

Fiber is used to reduce diarrhea in patients on enteral nutrition, but the effects on critically ill patients are moderate and non-conclusive, possibly due to the heterogeneity of the population and the different types of fiber [89]. A recent randomized controlled study among neuro-critical care patients compared the use of standard enteral formula versus enteric formula with prebiotic (fructooligosaccharides) content. The results showed that the patients on fiber-enriched diets achieved the target nutritional intake earlier compared to the group on standard enteral diets. Importantly, the probiotic-enriched diet also reduced the rate of diarrhea [90]. Nowadays, the American Society for Parenteral and Enteral Nutrition (ASPEN) recommends soluble fiber to treat diarrhea in hemodynamically stable critically ill patients [91].

The use of prebiotics in combination with probiotics, i.e., synbiotics, is limited in critically ill patients, with few published studies [92,93,94,95,96]. A meta-analysis conducted by Manzanares et al [97] analyzed the therapeutic efficacy of four trials with synbiotics versus 10 trials using probiotics, and found no additional benefit of the synbiotics compared to the probiotics alone.

#### 4.3.2. Use of Probiotics in Ventilator-Associated Pneumonia (VAP)

Ventilator-associated pneumonia (VAP) is responsible for up to 47% of the infections acquired in the ICU [98], and is one of the main causes of mortality and morbidity [99]. Many strategies have been described to prevent it, but the results are not conclusive [98]. Recently, numerous studies have focused on the use of probiotics [23,98,99].

The different meta-analyses that have been conducted provide similar conclusions. They show that the use of probiotics in critically ill patients decreases the incidence of VAP, but has no impact on the mortality, duration of mechanical ventilation, or length of ICU stay. They also report that clinical heterogeneity and potential publication bias decrease solid clinical recommendations, and suggest the need for additional high-quality clinical trials to be able to show significant evidence of the possible therapeutic effects of the probiotics [98,100].

Shimizu et al. [96] have also investigated the ability of a synbiotic (*Bifidobacterium breve* strain Yakult, *Lactobacillus casei* strain Shirota, and galactooligosaccharides) to reduce complications in VAP and modulate the gut microbiome, confirming a positive effect.

Despite the limitations of the different clinical trials conducted so far, there is an overall benefit derived from the use of probiotics in the prevention of VAP.

#### 4.3.3. Use of Probiotics in the Prevention of Antibiotic-Associated Diarrhea and *Clostridium Difficile* Infections

A meta-analysis that includes more than 11,800 patients showed a 40% reduction of antibiotic-associated diarrhea with the use of different probiotics (*Lactobacillus*, *Bifidobacterium*, *Saccharomyces*, *Streptococcus*, *Enterococcus*, and/or *Bacillus*) [101]. These results are supported by a meta-analysis published by Cochrane that concluded that the use of probiotics in colitis- and *C. difficile*-associated diarrhea reduced the incidence by 64% in patients who received antibiotics [102]. Besides, the side effects associated with antibiotics were also diminished.

#### 4.3.4. Use of Probiotics in Sepsis

A clinical trial focused on the benefit of probiotic and synbiotic therapy against sepsis in newborns reported a significant reduction in the combination of sepsis and death as the primary outcome of the study. These findings are important because they suggest that the majority of neonatal sepsis could be prevented using a synbiotic containing *Lactobacillus plantarum* ATCC-202195 [103]. Despite the possible differences in the infant and adult microbiome, these results raise the question of whether the prophylactic administration of a well-selected synbiotic therapy could prevent secondary sepsis in ICU patients.

Furthermore, it has been reported that *L. plantarum*, alone or in a multispecies mixture, is effective in reducing nosocomial infections in critically ill patients [97]. Thus, in 2015, the Canadian Clinical Practice Guidelines Committee stated that the use of probiotics should be considered in critically ill patients. As a wide range of species and doses of probiotics have been used in the different trials, no recommendation could be made for the dose or a particular type of probiotic, with the exception of *Saccharomyces boulardii*, which should not be used as it is considered unsafe in ICU patients [104]. In addition, a recent study showed that critically ill patients who were treated with probiotics had a greater risk of developing Lactobacillus bacteremia compared to non-treated patients [105]. Clearly, larger and better-designed clinical trials are needed to confirm the benefits of probiotics in sepsis before their use can be recommended in ICU patients.

#### 4.3.5. Fecal Microbiota Transplantation (FMT)

FMT has emerged as a beneficial therapy in ICU patients [106]. FMT has been implemented in the treatment and eradication of *C. difficile*, but it is increasingly used as a modulator of the microbiota in other pathologies such as inflammatory bowel disease, irritable bowel syndrome, obesity, metabolic syndrome, multiple sclerosis, Parkinson’s disease, and even chronic fatigue syndrome [107]. The experience of the use of FMT in critically ill patients is very limited [106,108,109,110]. A recent multi-center study in critically ill patients with antibiotic-associated diarrhea reported beneficial effects after FMT, including the reduction of abdominal pain, diarrhea, abdominal distension, and hematochezia [111]. Within the first days of the FMT, a reduction in fever and amelioration of the systemic inflammatory response syndrome (SIRS) was observed in all patients. A major problem limiting the efficacy of FMT in ICU patients is the prevalent use of antibiotics. Some authors have proposed stopping antibiotics before and after FMT so that the transplanted bacteria can reshape the microbiome [112]. Another major limitation is the risk that FMT poses in immunocompromised patients [113], where they can cause serious infections. Since in most studies and clinical trials using FMT, immunocompromised patients are excluded, no recommendations have been made for this patient group [114,115]. However, despite the above limitations/risks, there are several case series of successful FMT in immunocompromised patients [116,117,118]. Importantly, all these problems need to be resolved before this therapy can be administered to critically ill patients in ICUs.

## 5. Conclusions

In critically ill patients, the microbiome is compromised, resulting in a disease-promoting pathobiome. It is very important to characterize it properly to develop efficient nutritional therapies that can revert this state, especially enteral nutrition. Among these, oral probiotic supplements represent a logical approach to treat critical illness, since they can restore the intestinal microbiome and suppress the growth of pathogens, which could constitute the main source of septic infections. Furthermore, they also help in the recovery of systemic infection by attenuating inflammation and promoting organ function. Of note, the specific molecular mechanisms exerted by probiotics are not fully elucidated, and their beneficial effects are specific to particular species and possibly even subspecies or strains, and these therapeutic properties cannot be assumed for other species even within the same genus. Besides, it is necessary to follow up these patients for longer periods in order to monitor for reoccurring infections in time. This information is essential for the development of targeted therapies, such as the use of probiotics, or fecal microbiota transplantation, to restore the microbiome and prevent infections during the convalescence state of the patient.

## Figures and Tables

**Table 1:** Enteral Nutrition in special conditions.

Enteral Nutrition in Special Conditions		
Early EN should be implemented	Low dose EN should be administered	EN should be delayed
Patients receiving ECMO	Patients with therapeutic hypothermia	Patients with uncontrolled shock (when hemodynamic and tissue perfusion goals are not reached)
Patients with traumatic brain injury	Patients with intra-abdominal hypertension without abdominal compartment syndrome	Patients in uncontrolled life-threatening hypoxemia, hypercapnia or acidosis
Patients with stroke (ischemic or hemorrhagic)	Patients with acute liver failure	Patients suffering from active upper gastrointestinal bleeding
Patients with spinal cord injury		Patients with overt bowel ischemia
Patients with severe acute pancreatitis		Patients with high-output intestinal fistula
Patients after gastrointestinal surgery		Patients with abdominal compartment syndrome
Patients after abdominal aortic surgery		Patients with gastric aspirate volume above 500 mL/6 h.
Patients with abdominal trauma while the continuity of the gastrointestinal tract is restored		
Patients delivery neuromuscular blocking agents		
Patients managed in prone position		
Patients with an open abdomen		

Note: Recommendations published by the European Society of Intensive Medicine (ESCIM) for the initiation of early enteral nutrition (within 48 h of Intensive Care Unit admission) and recommendations favoring delaying it [63]. EN (enteral nutrition), ECMO (ExtraCorporeal Membrane Oxygenation).
